# Development and Application of Low-Cost and Eco-Sustainable Bio-Stimulant Containing a New Plant Growth-Promoting Strain *Kosakonia pseudosacchari* TL13

**DOI:** 10.3389/fmicb.2020.02044

**Published:** 2020-08-18

**Authors:** Ida Romano, Valeria Ventorino, Patrizia Ambrosino, Antonino Testa, Fatima Ezzahra Chouyia, Olimpia Pepe

**Affiliations:** ^1^Department of Agricultural Sciences, University of Naples Federico II, Naples, Italy; ^2^Agriges S.r.l. – Nutrizione Speciale per L’Agricoltura Biologica e Integrata, San Salvatore Telesino, Italy; ^3^Department of Biology, Faculty of Sciences and Techniques, Hassan II University of Casablanca, Casablanca, Morocco

**Keywords:** inoculant, PGPR, rhizocompetence, bio-formulate, organic by-products

## Abstract

The use of beneficial microbes as inoculants able to improve fitness, growth and health of plants also in stress conditions is an attractive low-cost and eco-friendly alternative strategy to harmful chemical inputs. Thirteen potential plant growth-promoting bacteria were isolated from the rhizosphere of wheat plants cultivated under drought stress and nitrogen deficiency. Among these, the two isolates TL8 and TL13 showed multiple plant growth promotion activities as production of indole-3-acetic acid (IAA), siderophores, ammonia, and 1-aminocyclopropane-1-carboxylic acid (ACC) deaminase production, the ability to solubilize phosphate as well as exerted antimicrobial activity against plant pathogens as *Botrytis* spp. and *Phytophthora* spp. The two selected strains were identified as *Kosakonia pseudosacchari* by sequencing of 16S rRNA gene. They resulted also tolerant to abiotic stress and were able to efficiently colonize plant roots as observed *in vitro* assay under fluorescence microscope. Based on the best PGP properties, the strain *K. pseudosacchari* TL13 was selected to develop a new microbial based formulate. A sustainable and environmentally friendly process for inoculant production was developed using agro-industrial by-products for microbial growth. Moreover, the application of *K. pseudosacchari* TL13- based formulates in pot experiment improved growth performance of maize plants.

## Introduction

According to the Food and Agriculture Organization (FAO), the estimated world population for 2025 will be nearly 8.5 × 10^9^ inhabitants (Timmusk et al., 2017). Such an increase in agricultural production of 60% within the next years could be required to satisfy global food demand (Berger et al., 2018). Actually, in order to maintain a high quality of agricultural productions and eliminate or minimize yield loss, chemicals (fertilizers, pesticides, herbicides, etc.), hormones and antibiotics are commonly used for crops. The use of agrochemicals at industrial level allows to produce a large number of agricultural products at low costs with high profits for farmers. However, serious concerns regarding human and environmental health resulting from chemical residues in soil, water and food as well as farm workers’ exposure have posed great attention (Alori and Babalola, 2018). Indeed, in the last two decades, the demand for organically grown agricultural products increased as consequence to the request for healthy and safe products (Dorais and Alsanius, 2016). Therefore, new eco-compatible strategies to improve agricultural systems and crop production are needed. The use of plant beneficial microorganisms as inoculants offers an attractive eco-friendly alternative strategy to chemical inputs to ensure crop yield and nutritional quality (Fiorentino et al., 2018) acting as agricultural probiotics. Probiotics are live microorganisms that offer benefits to the host providing nutritional inputs and protecting it from pathogens (Hossain et al., 2017). Among the beneficial microbes employed in agriculture, plant growth-promoting rhizobacteria (PGPR) are the most commonly used. These microbes are able by a wide range of mechanisms to improve nutrient availability in soil, plant nutrient uptake and assimilation [i.e., nitrogen fixation, phosphate solubilization, siderophore, indole-3-acetic acid (IAA), and ammonia production] and/or providing protection against plant pathogens (Backer et al., 2018; Woo and Pepe, 2018). Indeed, these microbes could also act as bio-stimulants ameliorating plant growth and crop production in response to abiotic stress in hostile environments (Viscardi et al., 2016; Van Oosten et al., 2017).

Important examples of PGPR include *Pseudomonas*, *Bacillus*, *Azotobacter*, *Azospirillum*, *Burkholderia*, *Sphingomonas* (Sharma et al., 2011; Singh et al., 2013; Castanheira et al., 2017; Dal Cortivo et al., 2017; Kandel et al., 2017; Khalid et al., 2017). However, in the last few years, it is rising the interest around the genus *Kosakonia* for its potential PGP activities. Recently, several members of this genus have been recognized as endophyte of different agricultural plants and it was demonstrated their growth-promoting effects and crop yield improvement (Kämpfer et al., 2016; Berger et al., 2017). However, this genus being relatively young, it is poorly studied and many of its features remain still unexplored.

Actually, the establishment of a low-cost and eco-sustainable process, as well as an effective and stable formulation, are among the main biotechnological challenges for the development of microbial inoculants. The use of agro-industrial organic waste and by-products as carbon source for the growth and production of microbial biomass is an attractive strategy to reduce the production costs, to valorize organic waste and by-products and to develop a sustainable and environmentally friendly process for inoculant production at industrial level. Moreover, it is also very important the form (solid or liquid) of microbial inoculant as well as its shelf-life. In fact, the form of the inoculant could influence its cost production, affect its efficiency and determine the method of application in agriculture on large scale (Alori and Babalola, 2018). The inoculant must be easy to handle in the field but it should maintain its features during the process and an adequate viability and shelf-life since it is required that it should be stable for at least 6 months (Berger et al., 2018).

This work aimed to isolate, select and characterize rhizobacteria with multiple PGP properties and antimicrobial activity. The selected plant growth-promoting bacteria (PGPB) strains were also tested for their ability to tolerate abiotic stress and to be able to efficiently colonize plant roots in *in vitro* experiments. Additionally, the selected strain was used to develop a new inoculant using agro-industrial by-products as sole carbon source for microbial growth and the new low-cost and eco-sustainable bio-formulates were finally tested in two forms (solid or liquid) in pot experiment to improve growth performance of maize plant.

## Materials and Methods

### Soil Sampling and Microbial Isolation

Rhizosphere samples were collected according to Romano et al. (2020) from wheat plants grown, under drought stress and nitrogen deficiency, in a greenhouse at the experimental station of the University of Naples Federico II (Bellizzi, Italy; 43°31′N, 14°58′E, 60 m a.s.l.). Ten grams of samples were shaken for 30 min in 90 mL of quarter strength Ringer’s solution (Oxoid, Milan, Italy) containing tetrasodium pyrophosphate (16% w/v) as previously described (Ventorino et al., 2012a). After shaking, tenfold serial dilutions (1:10) were performed and used to inoculate liquid Augier medium (Pepe et al., 2013) for the detection of free-living (N_2_)-fixing aerobic bacteria. After incubation for 14 days at 28°C, the brown rings formed by microorganisms grew in the liquid medium were used to inoculate LG agar medium (Aquilanti et al., 2004). The plates were incubated for 7 days at 28°C. Isolated colonies were picked from plates, purified by streaking on the same isolation medium, characterized by different morphologies examined by microscopy, gram staining and catalase reaction and stored at 4°C as slant cultures until their characterization.

### Preliminary Screening for Plant Growth Promoting Traits

Thirteen bacterial isolates were screened on the basis of their potential plant growth promotion activities. Detection and quantification of IAA production was determined by the Salkowski colorimetric assay using Nutrient Broth (Oxoid) with and without l-tryptophan (2 mg L^–1^; Sigma-Aldrich, Milan, Italy) as previously described (Ventorino et al., 2014).

Semi-quantitative agar spot method was used to determine the ability of bacterial isolates to produce siderophores by Chrome azurol S (CAS) assay as described by Silva-Stenico et al. (2005). After 14–21 days of incubation at 28°C, the formation of an orange or yellow halo around the colony indicated the production of siderophores by the microorganism.

Determination of 1-aminocyclopropane-1-carboxylic acid (ACC) deaminase activity of isolates was performed by assessing the growth on nitrogen-free minimal medium (MM) agar supplemented with 3 mM ACC (Sigma-Aldrich) after incubation at 28°C in the dark for 7 days as described by Jaemsaeng et al. (2018). MM agar supplemented with 2 g L^–1^ (NH_4_)_2_SO_4_ was used as control.

### Identification of Selected Strains

The bacterial isolates showing the highest plant growth promoting activities were selected for further investigations and identified by the sequencing of the 16S rRNA gene. In detail, total genomic DNA of selected strains was extracted by boiling for 10 min and then used as template for the PCR assay. The PCR mixture was employed according to Alfonzo et al. (2012) using the primers fD1 (5′-AGAGTTTGATCCTGGCTCAG-3′) and rD1 (5′-AAGGAGGTGATCCAGCC-3′). The PCR conditions were as described by Ventorino et al. (2017). PCR products were purified using the QIAquick PCR Purification Kit (Quiagen, Milan, Italy) according to the supplier’s recommendations and sequenced as previously reported (Ventorino et al., 2016). The DNA sequences were compared to the GenBank nucleotide data library using the BLAST software at the National Centre for Biotechnology Information website^1^.

The nearly full-length 16S rRNA sequences of the selected bacterial strain and 31 type strains belonging to different genera related to *Kosakonia pseudosacchari* species as described by Kämpfer et al. (2016) and Wang et al. (2019) were used to perform multiple nucleotide alignments using the ClustalW program (Thompson et al., 1994) from MEGA version X (Kumar et al., 2018; Stecher et al., 2020). The nucleotide sequences of the type strains were retrieved from the Ribosomal Database Project (RDP)^2^ and from the National Center for Biotechnology Information (NCBI)^3^. The phylogenetic tree was inferred using the Neighbor-Joining method with the Maximum Composite Likelihood model in the MEGAX program, with bootstrap values based on 1,000 replications.

The 16S rRNA gene sequences obtained from selected bacterial strains were deposited in the GenBank nucleotide database under accession numbers MN607213 and MN607214 (see footnote 3).

### Characterization of Selected Strains for PGP Traits and Antimicrobial Activity

Phosphate solubilization ability was quantified by molybdenum blue quantitative assay in Pikovskaya’s (PVK) liquid medium. Briefly, 10 mL of PVK medium was inoculated with 0.1 mL of bacterial cultures (approximately 1.5 × 10^8^ CFU mL^–1^) and incubated for 15 days at 30°C. After incubation, cultures were centrifuged (5 min at 18,620 × *g*) and supernatant was collected to estimate released soluble phosphorus (P) as described by Murphy and Riley (1962). The concentration of P solubilized was determined by spectroscopic absorbance measurements at 430 mμ according to the standard curve (Murphy and Riley, 1962).

Ammonia production of selected strains was estimated by inoculating the microorganisms in 5 mL of peptone water according to Cappuccino and Sherman (1987) and incubating under shaking (100 rpm) at 30°C for 7 days. The presence of ammonia was detected by the development of a brown to yellow color after adding 0.5 mL of Nessler’s reagent (Sigma-Aldrich) to the culture and then quantified by spectroscopic absorbance measurements at 420 nm according to the standard curve (Passari et al., 2017).

The presence of the target gene *nif*H, encoding nitrogenase reductase enzyme, was assessed by PCR assay using the synthetic oligonucleotide primers *nif*H-F (5′-AAAGGYGGWAT CGGYAARTCCACCAC-3′; Rösch et al., 2002) and *nif*H-R, (5′-TTGTTSGCSGCRTACATSGCCATCAT-3′; Rösch et al., 2002) using conditions reported by Fiorentino et al. (2016). The presence of the target gene was assessed by visualization of a 475 bp band by agarose (1.5% w/v) gel electrophoresis (100 V for about 1 h).

A 1-aminocyclopropane-1-carboxylic acid deaminase activity was quantified according to Penrose and Glick (2003) by measuring the amount of α-ketobutyrate (Sigma-Aldrich) produced when the enzyme ACC deaminase cleaves ACC. In detail, bacterial strains were inoculated in 5 mL of Dworkin and Foster (DF) minimal salt medium containing (NH_4_)_2_SO_4_ as sole nitrogen source (Penrose and Glick, 2003). After incubation at 30°C for 48 h, the cultures were used to inoculate 5 mL of DF salt medium containing 3 mM ACC (Oxoid) as nitrogen source. The amount of α-ketobutyrate (μmol) produced was estimated by measuring the absorbance at 540 nm according to the standard curve (α-ketobutyrate concentration ranged from 0.1 to 100 μmol).

Quantitative estimation of siderophores was performed according to Arora and Verma (2017) using CAS reagent and expresses as percent siderophore unit (psu) using the following formula (Payne, 1993):

psu=[(Ar-As)⁢x⁢100]/Ar

where Ar is the absorbance of reference (CAS solution and uninoculated medium), and As is the absorbance of sample (CAS solution and cell-free sample supernatant).

Antimicrobial antagonism was evaluated using the dual culture method described by Hammami et al. (2013) against eight pathogenic eukaryotic strains belonging to the microbial collection of Division of Biology and Protection of Agricultural and Forest Systems (Department of Agricultural Sciences, University of Naples Federico II): *Botrytis cinerea* B11, *B. cinerea* B12, *Fusarium oxysporum* F3, *F. oxysporum* F5, *Aspergillus niger* A31, *Phytophthora infestans* ph1, *Phytophthora cactorum* ph3, and *Phytophthora cryptogea* ph4. Fungi were grown on Potato Dextrose Agar (PDA, Oxoid) at 28°C for 7 days, while Oomycetes were grown on V8 agar (200 mL of V8 juice, 2.5 g CaCO_3_, 800 mL of distilled water and 17 g of bacteriological agar) at 28°C for 21 days. Conidia were harvested from the surface of plates by flooding the cultures with 9 mL of sterilized distilled water and gently scraping with a sterilized glass rod. The conidial concentration was determined using the counting chamber Thoma (Hawksley, United Kingdom). An over-layer agar (agar 0.7%) containing a concentration of 10^5^ conidia mL^–1^ of each plant pathogen was poured on Brain Heart Infusion (BHI) agar plates previously spotted with the bacterial strains. After incubation for 7 or 21 days at 28°C, the antimicrobial activity of the bacterial strains was highlighted by the presence of a halo around the colony without fungal growth.

### Tolerance to Abiotic Stress

The two selected bacterial strains were tested for their salt tolerance in liquid medium as previously described Ventorino et al. (2012b). Briefly, 5 mL of BHI medium supplemented with NaCl up to 15% (w/v) was inoculated with each bacterial strain. The standard BHI medium with 0.5% (w/v) NaCl was used as control. Bacterial growth was determined by observing the development of turbidity of cultures at 24 and 48 h and comparing them with McFarland Turbidity Standard.

Similarly, temperature tolerance was investigated comparing bacterial growth in BHI liquid medium with McFarland Turbidity Standard after 24 and 48 h of incubation at 28, 30, 37, and 42°C.

Finally, pH tolerance was determined by evaluating the growth of bacterial strains in BHI liquid medium in which pH was adjusted at pH 5, 6, 7, and 8 by the addition of HCl or NaOH. After incubation, bacterial growth was estimated at 24 and 48 h comparing their turbidity to McFarland Turbidity Standard.

### Rhizosphere Competence

Tomato seeds (*Solanum lycopersicum* var. *cerasiforme*) were carefully de-husked without damaging the embryo and surface sterilized as described by Banik et al. (2016). Briefly, seeds were treated with 2% sodium hypochlorite (5 min) followed by washing with sterile distilled water, then seeds were treated with 75% ethanol (5 min), washed again with sterile water and treated with 30% hydrogen peroxide (2 min) as suggested by Amarasinghe et al. (2018); finally, they were carefully rinsed ten times with sterile distilled water. Seeds sterility was checked by plating on Plate Count Agar (PCA; Oxoid). Seeds germination took place in darkness at 30 ± 2°C.

Microbial cells were grown in BHI medium (30 ± 2°C, 24 h). Cells were harvested by centrifugation (2000 × *g* for 5 min) at the end of their exponential phase of growth, washed twice in HEPES buffer (0.1 M) and then suspended in quarter strength Ringer’s solution (Oxoid) until achieving microbial concentration of approximately 5 × 10^8^ CFU mL^–1^ (counting chamber Thoma 0.02 depth, Hawksley United Kingdom). Finally, tomato seedlings were treated with bacterial suspension for 48 h at 30 ± 2°C and then rinsed five times with sterile HEPES buffer (0.1 M) to remove the loosely associated bacteria from the radicle surface. Tomato seedlings treated with sterile water were used as control. Bacteria-infected radicles and controls were treated with LIVE/DEAD^®^ BacLight^TM^ bacterial viability kit (Thermo Fisher Scientific) following the manufacturer’s instructions. Treated radicles were observed by fluorescence microscope (Axiovert 200M, Zeiss, Göttingen, Germany) under UV light (50-W mercury lamp) and using a Green Fluorescent Protein Filter (38 HE-GFP; excitation wavelength of 450–490 nm) and Rhodamine Filter (Rh-20; excitation wavelength of 540–552 nm).

### Production of a Low-Cost Bacterial Inoculants

#### Study of Bacterial Growth

The strain TL13 was inoculated in 200 μL of BHI using 96-well flat-bottom microplate in a Microplate Reader (BioTek Elx808) and incubated at 30°C for 24 h with moderate shaking every 30 min. O.D_600nm_ measurements were performed every 30 min to define the growth curve.

Preliminary batch growth tests were performed to assess the best growth conditions for the strain TL13. In details, 500 mL flasks filled with BHI medium were inoculated with 2% bacterial cells suspension (8.45 ± 0.20 CFU mL^–1^) and incubated at 30°C for 24 h using three different growth conditions: (1) Batch 1, shaking at 130 rpm (Grant-bio, Orbital Shaker-Incubator ES80); (2) Batch 2, shaking at 130 rpm and sterile air sparging at 0.5 vvm; control, no shaking and no air sparging. Samples were withdrawn every 2 h and cell growth was determined by viable counting on BHI medium.

A scale-up batch experiment was performed in a 14 L fermentor (New Brunswick BioFlo^®^/CelliGen^®^ 115, Eppendorf) to evaluate the microbial growth using the best conditions assessed in the preliminary batch experiments. The experiment was performed in a working volume of 4 L of BHI medium inoculated with 2% bacterial cells suspension (8.67 ± 0.40 CFU mL^–1^), using the following parameters: 30°C, pH 7.00, agitation of 130 rpm, air sparging at 0.5 vvm, 40 mL of a solution 3% of Antifoam 204 (Sigma-Aldrich) added at the beginning of the process. Samples were withdrawn every 2 h and cell growth was determined by viable counting on BHI medium. After 24 h, the culture was centrifuged (45 min at 3428 × *g*) and recovered cells were suspended in a 5% sucrose solution at the ratio 1:5 (w:v). The strain was freeze-dried, and cell viability was determined by counting on BHI medium immediately after freeze-drying and after 3 and 6 months of storage at room temperature.

#### Microbial Growth in Liquid Media Containing Food By-Products

The strain TL13 was inoculated in several liquid media containing agro-food industrial by-products to find a low-cost carbon source useful for its growth. To this end, the strain was inoculated into 10 mL of liquid substrates containing 1, 5 or 10% of whey, protein hydrolysate, exhausted yeasts, molasse or vinasse, kindly provided by Agriges S.r.l. (San Salvatore Telesino, Benevento, Italy). The strain TL13 grown in BHI was used as control. Samples were withdrawn after 48 h of incubation at 30°C, to determine bacterial growth.

#### Production of Bacterial Inoculants on Nutrient-Supplemented Vermiculite

Solid state fermentation (SSF) was performed in gas permeable polypropylene bags (SacO2, Belgium). Growth on inert support was carried out by adopting the procedures described by Graham-Weiss et al. (1987). Sterile vermiculite, moistened with BHI broth or with a solution of exhausted yeasts and vinasse, was inoculated with the selected strain TL13 (10^6^ bacterial cells per g of vermiculite). After incubation (15 days at 30 ± 1°C), an aliquot was used to develop liquid inoculants recovering the bacterial cells and added them in a raw castor oil/alginate based emulsion following the protocol described by Fravel et al. (1985) with some modifications. Another amount of inoculated vermiculite was dried for 15 days at 30 ± 2°C to achieve a microbial-based solid formulation. Samples were withdrawn immediately after incubation and after the development of formulations to determine bacterial growth by viable counting on BHI medium.

#### Pot Trials

The ability of the selected strain TL13 to promote plant growth was evaluated in growth chamber pot trials. The experimental set up was performed according to standard procedure (DM 27/01/2014, 2014) with some modifications. Maize (*Zea mays*, Class FAO 400/gg 120) seeds were surface sterilized by 5 min washing in NaClO 5% solution and germinated on damp tissue paper for 48 h. Seeds were planted in 10 cm Ø plastic pots filled with 0.5 kg of unsterilized soil (35% clay, 27% silt and 38% sand; pH-H_2_O 7.7; electrical conductivity 0.6 dS m^–1^; CaCO_3_ 37 g kg^–1^; organic matter 16.3 g kg^–1^; organic carbon 9.5 g kg^–1^; total nitrogen 1.2 g kg^–1^; C/N ratio 7.9; phosphorus available 59 mg kg^–1^; potassium exchangeable 296 mg kg^–1^; calcium exchangeable 2089 mg kg^–1^; magnesium exchangeable 111 mg kg^–1^; sodium exchangeable 57 mg kg^–1^; Cation Exchange Capacity 12.1 cmol(+) kg^–1^). At planting, soil was inoculated with the strain TL13 at a concentration of approximately 1 × 10^6^ cells g^–1^.

The strain TL13 was inoculated in three different formulates: raw castor oil/alginate based emulsion (E-TL13), dried vermiculite (V-TL13) and recovered cells (R-TL13) diluted in sterile Ringer’s solution (Oxoid). Un-inoculated soil (C) was used as control. All tests were performed in triplicate and three seeds were planted for each pot.

Plants were grown under controlled conditions with a constant temperature of 28 ± 0.5°C, a 16 h light/8 h dark photoperiod, relative moisture 70% and daily watered for 15 days.

After 15 days, the plants were sampled and were measured vegetative parameters as total plant length, root and shoot length, root and shoot fresh weight, root and shoot dry weight percentage.

### Statistical Analyses

Data were analyzed by one-way ANOVA followed by Duncan’s HSD *post hoc* for pairwise comparison of means (at *P* < 0.05) using SPSS 19.0 statistical software package (SPSS Inc., Cary, NC, United States).

## Results

### Plant Growth Promoting Activities of Bacterial Isolates

A total of 13 bacterial isolates (from TL1 to TL13) were obtained from the rhizosphere of wheat plants using Augier liquid medium followed by streaking on LG agar medium. Isolates were preliminarily screened for their potential plant growth promoting activities, as IAA and siderophores production and ACC-deaminase activity (Table 1).

**TABLE 1:** Preliminary screening for the assessing the plant growth-promoting activities of bacterial isolates obtained from wheat rhizosphere.

Isolate	IAA^†^ in NB (mg L^–1^)	IAA^§^ in NB + TRP (mg L^–1^)	Siderophores^#^ (mm)	ACC-deaminase activity^∗^
TL1	2.59 ± 0.06^*g*–*m*^	0.00 ± 0.00^*n*^	20.0 ± 0.00^*cd*^	++
TL2	3.16 ± 0.50^*g*–*i*^	1.82 ± 0.31^*i*–*m*^	0.00 ± 0.00^*f*^	+
TL3	1.56 ± 0.07^*l*–*n*^	1.32 ± 0.02^*mn*^	30.0 ± 0.00^*ab*^	−
TL4	3.40 ± 0.02^*f*–*h*^	4.90 ± 0.63^*de*^	33.33 ± 5.77^*a*^	+
TL5	1.62 ± 0.02^*i*–*m*^	1.42 ± 0.00^*mn*^	23.33 ± 5.77^*bc*^	−
TL6	5.98 ± 1.03^*d*^	3.95 ± 0.33^*e*–*g*^	10.0 ± 0.00^*e*^	++
TL7	5.89 ± 0.60^*d*^	5.15 ± 0.51^*de*^	23.33 ± 5.77^*bc*^	++
TL8	13.20 ± 1.80^*c*^	12.91 ± 0.64^*c*^	23.33 ± 11.55^*bc*^	++
TL9	0.00 ± 0.00^*n*^	0.00 ± 0.00^*n*^	0.00 ± 0.00^*f*^	−
TL10	1.68 ± 0.67^*i*–*m*^	4.69 ± 1.32^*d*–*f*^	0.00 ± 0.00^*f*^	−
TL11	2.23 ± 0.02^*h*–*m*^	0.00 ± 0.00^*n*^	13.33 ± 5.77^*e*^	−
TL12	1.43 ± 0.02^*mn*^	3.05 ± 0.07^*g*–*l*^	20.0 ± 0.00^*cd*^	−
TL13	22.16 ± 2.67^*b*^	33.26 ± 1.67^*a*^	30.0 ± 0.00^*ab*^	++

*^†^IAA production in Nutrient Broth without L-tryptophan, values represent the means ± SD of three replicates. ^§^IAA production in Nutrient Broth supplemented with L-tryptophan, values represent the means ± SD of three replicates. ^#^Halo size (mm) = diameter of clearing or halo zone/colony diameter, values represent the means ± SD of three replicates. Different letters after values indicate significant differences (P < 0.05). ^∗^− no growth; + middle growth; ++ high growth.*

The results indicated that about 85% of isolates were able to synthetize IAA, although most of them at low amounts (ranging from 1.32 to 5.98 mg L^–1^). The two isolates, TL8 and TL13, showed the highest IAA production up to 13.20 ± 1.80 or 22.16 ± 2.67 and 12.91 ± 0.64 or 33.26 ± 1.67 mg L^–1^, respectively, in the absence and in the presence of L-tryptophan (Table 1).

Ten isolates produced siderophores showing orange haloes around the colony in CAS agar ranging from 10 to 35 mm (Table 1). Among these, the isolates TL3, TL4, and TL13 exhibited the highest siderophores production (halo dimension 30–33 mm); while, the isolates TL1, TL3, TL7, TL8, and TL12 produced haloes ranging approximately from 20 to 23 mm.

Moreover, seven isolates (TL1, TL2, TL4, TL6, TL7, TL8, and TL13), corresponding to about 54%, revealed ACC-deaminase activity because they were able to grow on MM medium supplemented with ACC (Table 1).

### Identification and Phylogenetic Analysis of Selected Strains

**FIGURE 1:**
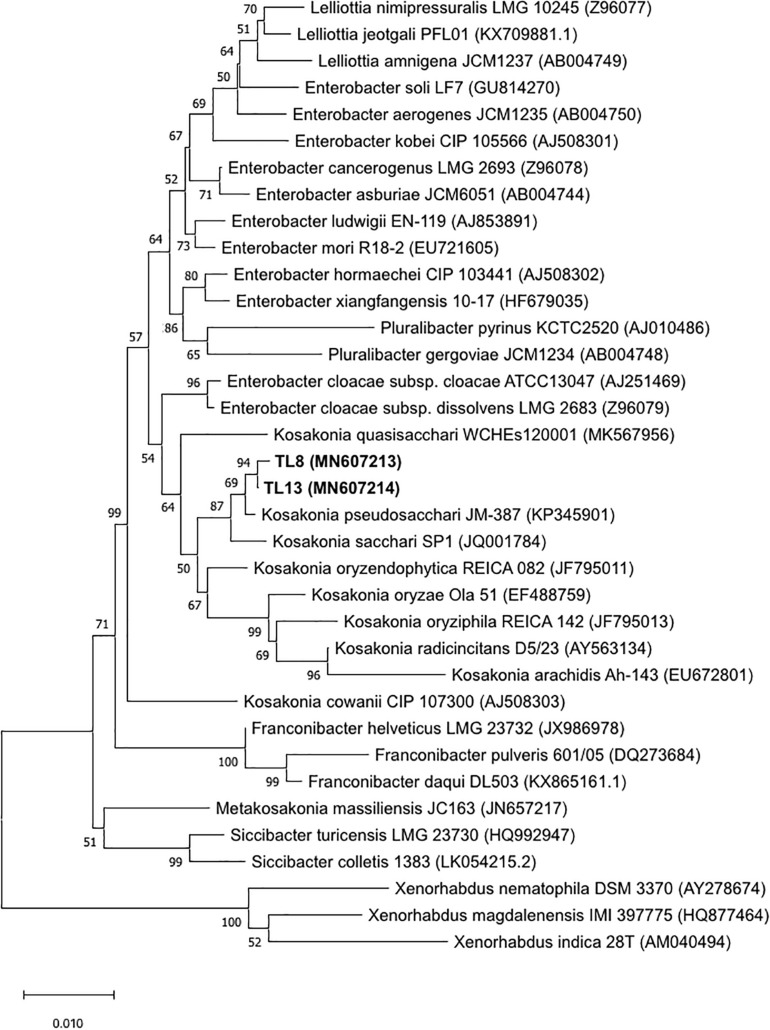
Neighbor-Joining tree based on the comparison of 16S rRNA gene sequences of bacterial strains TL8 and TL13 and 31 type strains related to genus *Kosakonia* sequences from RDP and NCBI. Bootstrap values (expressed as percentages of 1,000 replications) are given at the nodes. The sequence accession numbers used for the phylogenetic analysis are shown in parentheses following the species name. *Xenorhabdus* type strain sequences were used as out group. The scale bar estimates the number of substitutions per site.

The preliminary screening for the assessment of plant growth promotion activities allowed for the selection of the TL8 and TL13 isolates. The nearly full-length sequence of 16S rRNA gene (about 1,450 bp) of the strains TL8 and TL13 revealed an identity of 99% with *Kosakonia sacchari*, *K. pseudosacchari*, *Kosakonia oryzae*, and *Kosakonia radicincitans* species using Blast software. To establish the identification of the two selected strains, a phylogenetic tree generated from the distance data using the Neighbor-Joining method with the Maximum Composite Likelihood model in the MEGAX Program was constructed including the 16S rRNA sequences of type strains related to *Kosakonia* genus (Figure 1). High bootstrap values, ranging from 50 to 100%, were observed and indicated significant branching points in the phylogenetic tree. The phylogenetic tree indicated that the closest relative species of the two selected strains was *K. pseudosacchari* (cluster with bootstrap value of 69%), demonstrating that the strains TL8 and TL13 can be classified as belonging to this species (Figure 1).

### PGP Traits, Phenotypic Characteristics and Rhizosphere Competence of *Kosakonia pseudosacchari* Strains

The selected strains *K. pseudosacchari* TL8 and TL13 were further characterized to evaluate other plant growth promotion activities as well as antagonistic behaviors. Quantitative estimation of phosphate solubilization by molybdenum blue assay in PKV liquid medium indicated that the strains TL8 and TL13 were able to solubilize up to 348.05 ± 12.77 and 346.05 ± 25.62 mg L^–1^ of phosphate starting from dicalcium phosphate (Table 2). Measurement of ammonia in peptone water liquid medium by quantitative Nessler’s reagent test highlighted that both bacterial strains TL8 and TL13 were capable to produce ammonia (2.24 ± 0.03 and 2.37 ± 0.03 mg L^–1^, respectively; Table 2) in medium without nitrogen source. Moreover, *K. pseudosacchari* TL8 and *K. pseudosacchari* TL13 were potentially able to fix atmospheric nitrogen (N_2_) due to the presence of the *nif*H gene detected by specific PCR amplification as well as exhibited ACC deaminase activity producing up to 3.04 ± 0.10 and 3.31 ± 0.11 μM of α-ketobutyrate protein mg^–1^ in 30 min (Table 2). As reported in Table 2, quantitative assay showed a siderophore concentration produced by the strains *K. pseudosacchari* TL8 and TL13 equal to 32.00 ± 0.92 and 29.77 ± 1.8 psu, respectively. Indeed, both strains exerted antimicrobial activity against soil-borne plant pathogens (Table 2) revealed by a considerable reduction of mycelium growth of *B. cinerea* B12, *P. infestans* ph1, *P. cactorum* ph3, and *P. cryptogea* ph4, in respect to the control plates.

**TABLE 2:** Differential phenotypic characteristics and plant growth-promoting traits of bacterial strains *Kosakonia pseudosacchari* TL8 and TL13.

Characteristic/Activity	*Kosakonia pseudosacchari* TL8	*Kosakonia pseudosacchari* TL13
IAA in NB^†^ (mg L^–1^)	13.20 ± 1.80	22.16 ± 2.67
IAA in NB + T^§^ (mg L^–1^)	12.91 ± 0.64	33.26 ± 1.67
Siderophores production (psu)	32.00 ± 0.92	29.77 ± 1.8
ACC-deaminase activity (μM of α-ketobutyrate protein mg^–1^ in 30 min)	3.04 ± 0.10	3.31 ± 0.11
Ca_2_HPO_4_ solubilization (mg L^–1^)	348.05 ± 12.77	346.05 ± 25.62
*Nif*H gene	+	+
Ammonia accumulation (mg L^–1^)	2.24 ± 0.03	2.37 ± 0.03
NaCl tolerance range (w/v, 0.5–8%) 24 h	1 × 10^8^ CFU mL^–1^	1 × 10^8^ CFU mL^–1^
NaCl tolerance range (w/v, 9–13%) 48 h	1 × 10^6^ CFU mL^–1^	1 × 10^6^ CFU mL^–1^
pH range at 24 h	5–8	5–8
Temperature range (°C) at 24 h	28–42	28–42
Antagonistic activity	+ against *Botrytis cinerea* B12, *Phytophthora infestans* ph1, *Phytophthora cactorum* ph3, *Phytophthora cryptogea* ph4	+ against *Botrytis cinerea* B12, *Phytophthora infestans* ph1, *Phytophthora cactorum* ph3, *Phytophthora cryptogea* ph4

*^†^NB = Nutrient Broth. ^§^NB + T = Nutrient Broth supplemented with L-tryptophan.*

*Kosakonia pseudosacchari* TL8 and TL13 were found to be salt-tolerant because they were able to grow in the liquid culture medium containing up to 13% w/v of NaCl (Table 2). In detail, no differences were found in the bacterial growth up to 8.0% w/v of NaCl reaching a concentration of about 1 × 10^8^ CFU mL^–1^ after 24 h of incubation (Table 2). At higher NaCl concentration (from 9.0 to 13% w/v) the two strains grew slowly reaching a bacterial growth of two orders of magnitude lower (about 1 × 10^6^ CFU mL^–1^) after 48 h of incubation (Table 2). The two strains *K. pseudosacchari* TL8 and TL13 grew also up to about 1 × 10^8^ CFU mL^–1^ after 24 h of incubation at different temperatures (28, 30, 37, and 42°C). Finally, both strains tolerated a pH range between 4.0 and 8.0 reaching a final concentration of about 1 × 10^8^ CFU mL^–1^ after 24 h (Table 2).

**FIGURE 2:**
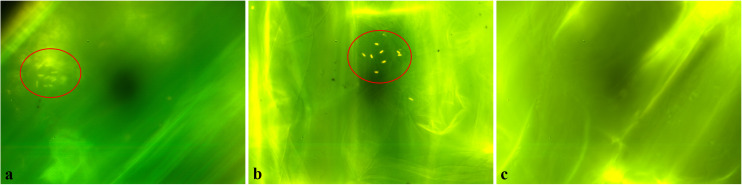
Colonization of tomato’s radicles by *Kosakonia pseudosacchari* TL8 **(a)**, *Kosakonia pseudosacchari* TL13 **(b)**, and uninoculated control **(c)** detected by LIVE/DEAD BacLigh bacterial viability kit and observed under fluorescence microscope.

In order to test the ability of the two selected strains *K. pseudosacchari* strains TL8 and TL13 to colonize the root surface, sterile tomato radicles were inoculated and observed by fluorescence microscope after staining with the LIVE/DEAD^®^ BacLight^TM^ kit reagents. As shown in Figure 2, bacterial cells were clearly visualized on plant tissues highlighting that both *K. pseudosacchari* TL8 and *K. pseudosacchari* TL13 successfully colonized tomato’s radicle. In particular, bacterial cells of the strains TL8 resulted congregated on root surfaces (Figure 2a); whereas, cells of the strain TL13 appeared scattered (Figure 2b).

### Investigation and Optimization of Growth Conditions

On the basis of PGP traits, the strain *K. pseudosacchari* TL13 was selected for further investigations in order to produce an innovative bacterial inoculant.

The first step was to explore and define the best growth conditions of the strain *K. pseudosacchari* TL13. To this end, a kinetic growth curve of the strain TL13 was obtained by Microplate Reader test. This preliminary investigation showed that the exponential phase started after 4 h of incubation and continued until 10 h, when begun the stationary phases (data not shown).

**FIGURE 3:**
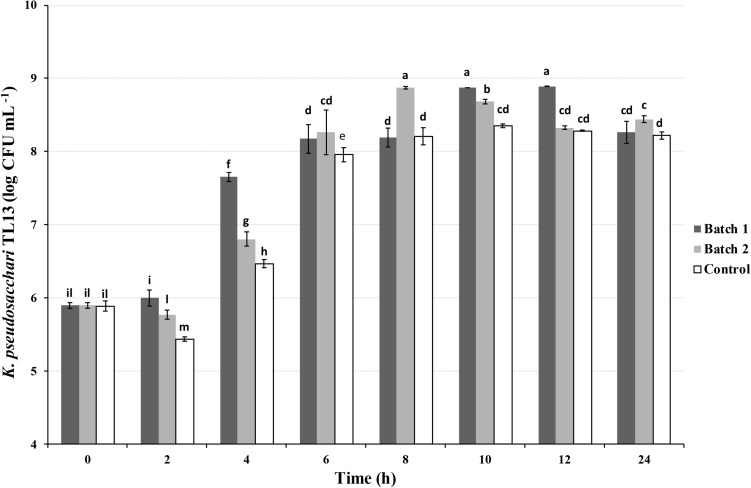
Viable count of *Kosakonia pseudosacchari* TL13 during its growth in batch experiments using BHI medium (30°C and pH 7.00). Batch 1: shaking at 130 rpm; Batch 2: shaking at 130 rpm and air sparging at 0.5 vvm; Control: no shaking and no air sparging. The error bars represent the means ± SD of three replicates. Different letters indicate significant differences (*P* < 0.05).

Batch experiments were then performed to investigate the effect of agitation and air sparging on the bacterial growth. The highest bacterial concentration in the shorter time was recorded in the batch 2 reaching a value of 8.87 ± 0.02 log CFU mL^–1^ after 8 h of incubation (Figure 3), after that, a significant decrease in its concentration was observed. Similarly, in the batch 1 was detected an increase of three orders of magnitude at 10 and 12 h (8.88 ± 0.00 log CFU mL^–1^ and 8.89 ± 0.00 log CFU mL^–1^) in respect to the beginning of the experiment (0 h; 5.90 ± 0.04 log CFU mL^–1^), decreasing up to 8.26 ± 0.15 log CFU mL^–1^ at 24 h (Figure 3). However, in both conditions, the *K. pseudosacchari* TL13 load was approximately one order of magnitude greater than that recovered in the control at the same sampling time (ranging from 5.89 ± 0.07 to 8.35 ± 0.03 log CFU mL^–1^; Figure 3).

**FIGURE 4:**
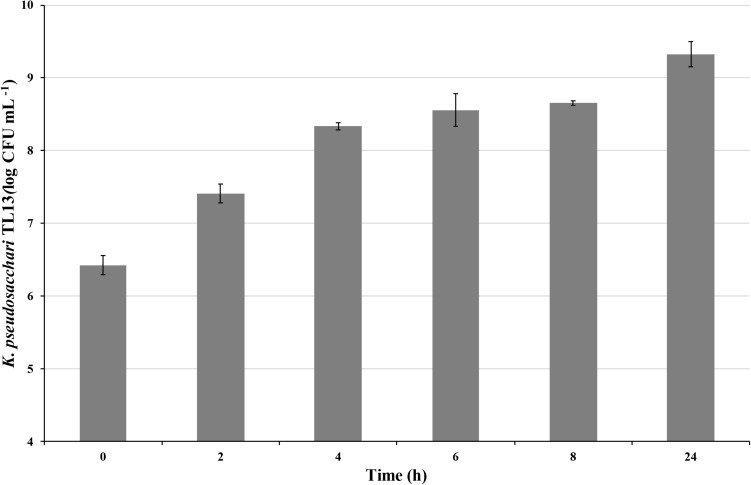
Viable count of *Kosakonia pseudosacchari* TL13 during its growth in 10 L fermentor at 30°C, pH 7.00, shaking at 130 rpm and air sparging at 0.5 vvm. The error bars represent the means ± SD of two replicates.

On the basis of these results, growth conditions of batch 2 (shaking at 130 rpm and air sparging at 0.5 vvm) were chosen to perform the scale-up of the experiment in a 10 L fermentor. In this condition, although at 8 h was detected a bacterial concentration (8.66 ± 0.02 log CFU mL^–1^) similar to that recorded in the previous batch experiment, the exponential phase persisted up to 24 h reaching a bacterial load of 9.33 ± 0.18 log CFU mL^–1^ (Figure 4). Moreover, to explore the tolerance of the strain *K. pseudosacchari* TL13 to desiccation and to test its shelf-life, the viability of freeze-dried bacterial cells obtained by fermentor experiment was estimated over time. Immediately after freeze-drying, a bacterial concentration of 10.43 ± 0.10 log CFU g^–1^ was determined. This value remained constant after 3 months of storage (10.40 ± 0.06 log CFU g^–1^) and decrease of about 1 log after 6 months reaching a concentration of 9.57 ± 0.14 log CFU g^–1^.

### Production of Eco-Friendly and Low-Cost Bacterial Inoculants

Different agro-food industrial by-products were used to obtain an eco-sustainable and cheap carbon source for the growth at industrial level of *K. pseudosacchari* TL13 and its use as inoculant. The strain TL13 resulted able to grow in presence of several carbon sources (whey, protein hydrolysate, exhausted yeasts, or vinasse) at different concentrations (1, 5 and 10%) reaching a bacterial load of about 8–9 log CFU mL^–1^ (Table 3). The only exception was the liquid medium containing molasse that determined the lowest bacterial growth at 5% (7.08 ± 0.18 log CFU mL^–1^) and no growth at 1 and 10% (Table 3). The highest bacterial growth was detected in the medium containing 10% exhausted yeasts (8.86 ± 0.21 log CFU mL^–1^), that was comparable to the optimal synthetic medium used as control (8.93 ± 0.01 log CFU mL^–1^), followed by the liquid medium containing 5% vinasse (8.81 ± 0.07 log CFU mL^–1^; Table 3). Therefore, SSF on nutrient-supplemented vermiculite of the strain *K. pseudosacchari* TL13 for the production of inoculant was performed using a solution of exhausted yeasts and vinasse. Microbial concentration increased after 15 days of incubation of about three orders of magnitude from 6.81 ± 0.05 to 9.34 ± 0.11 log CFU g^–1^. No significant differences (*P* > 0.05) were detected between *K. pseudosacchari* TL13 grown on vermiculite moistened with exhausted yeasts and vinasse and the vermiculite moistened with BHI used as control (9.20 ± 0.65 log CFU g^–1^).

**TABLE 3:** Viable counts of *Kosakonia pseudosacchari* TL13 (log CFU mL^–1^) after 48 h of growth at 30°C in several liquid media containing different agro-industrial by-products as carbon source at three percentage (1, 5, and 10%).

	Percentage of by-products in the liquid medium		
	
Agro-industrial			
by-products	1%	5%	10%
Whey	8.18 ± 0.02^*e*^	7.98 ± 0.07^*fg*^	7.95 ± 0.03^*fg*^
Protein hydrolysate	8.05 ± 0.01^*f*^	8.30 ± 0.03^*d*^	8.18 ± 0.07^*e*^
Exhausted Yeast	8.30 ± 0.09^*d*^	8.48 ± 0.08^*c*^	8.86 ± 0.21^*ab*^
Molasse	0.00 ± 0.00^*i*^	7.08 ± 0.18^*h*^	0.00 ± 0.00^*i*^
Vinasse	7.94 ± 0.02^*g*^	8.81 ± 0.07^*b*^	7.98 ± 0.02^*fg*^
BHI (control)	–	8.93 ± 0.01^*a*^	–

*Values represent the means ± SD of three replicates. Different letters indicate significant differences (*P* < 0.05).*

Solid state fermentation products were used to develop solid and liquid inoculants containing a microbial load of about 6.7–6.9 log CFU g^–1^ or mL^–1^, which remained constant up to 28 days.

## *In vivo* Pot Experiments

Maize plants were positively affected by inoculation with the strain *K. pseudosacchari* TL13. Indeed, several plant growth parameters significantly increased in the soils treated with solid or liquid inoculants as shown in Table 4. In particular, E-TL13 treatment (raw castor oil/alginate based emulsion containing *K. pseudosacchari* TL13 cells) showed the best results, in which a significant increase (*P* < 0.05) of total plant length (63.83 ± 4.51 cm), root length (23.67 ± 2.57 cm), and root fresh weight (1.28 ± 0.11 g) was recorded in E-TL13 treated plants in respect to the un-inoculated control (49.17 ± 3.40 cm, 11.06 ± 0.90 cm, and 0.80 ± 0.13 g, respectively; Table 4). Similarly, a significant increase in the root length was also observed in the V-TL13 (dried vermiculite containing *K. pseudosacchari* TL13 cells) and R-TL13 (*K. pseudosacchari* TL13 cells diluted in sterile Ringer’s solution) treatments reaching values of 18.50 ± 2.26 and 23.13 ± 1.99 cm, respectively (Table 4). Interestingly, V-TL13 treatment induced a significant increase of shoot dry weight percentage (9.62 ± 0.29%) compared to un-inoculated control (6.84 ± 0.40%; Table 4). However, also E-TL13 and R-TL13 treatments showed a similar trend of this plant parameter although no significant differences were detected (*P* > 0.05; Table 4).

**TABLE 4:** Effect of different inoculant formulations on total plant length (cm), root length (cm), shoot length (cm), root fresh weight (g), shoot fresh weight (g), root dry weight (%), shoot dry weight (%) of maize plants.

	Soil treatment			
	
Plant parameters	V-TL13	E-TL13	R-TL13	C
Total plant length (cm)	51.50 ± 4.63^*ab*^	63.83 ± 4.5^*a*^	58.56 ± 4.78^*ab*^	49.17 ± 3.40^*b*^
Root length (cm)	18.50 ± 2.26^*ab*^	23.67 ± 2.57^*a*^	23.13 ± 1.99^*a*^	11.06 ± 0.90^*b*^
Shoot length (cm)	33.00 ± 2.87^*a*^	40.17 ± 2.55^*a*^	35.44 ± 3.68^*a*^	38.11 ± 2.77^*a*^
Root fresh weight (g)	1.01 ± 0.15^*ab*^	1.28 ± 0.11^*a*^	0.81 ± 0.15^*b*^	0.80 ± 0.13^*b*^
Shoot fresh weight (g)	1.19 ± 0.21^*a*^	1.58 ± 0.14^*a*^	1.54 ± 0.30^*a*^	1.30 ± 0.27^*a*^
Root dry weight (%)	15.30 ± 1.64^*a*^	15.03 ± 1.52^*a*^	19.73 ± 1.08^*a*^	17.25 ± 2.42^*a*^
Shoot dry weight (%)	9.62 ± 0.29^*a*^	7.75 ± 0.64^*ab*^	8.53 ± 0.83^*ab*^	6.84 ± 0.40^*b*^

*V-TL13, soil inoculated with dried vermiculite containing *K. pseudosacchari* TL13; E-TL13, soil inoculated with raw castor oil/alginate based emulsion containing *K. pseudosacchari* TL13; R-TL13, soil inoculated with *K. pseudosacchari* TL13 cells diluted in sterile Ringer’s solution; C, un-inoculated soil. Values represent the means ± SD of three replicates. Different letters indicate significant differences (*P* < 0.05).*

## Discussion

### PGP Traits, Phenotypic Characteristics and Rhizosphere Competence

In the last decades, the development and the use of microbial inoculants have elicited great interest as an ecofriendly alternative strategy to the application of synthetic fertilizers for plant growth promotion and pest management. This approach improves the sustainability of agricultural systems by reducing environmental and human health risks due to the application of chemical fertilizers and pesticides in crop production (Rahman et al., 2018). In this context, it is necessary to find new microorganisms that can exert multiple plant beneficial activities to develop a low-cost inoculant. The ecological approach developed in this study enabled the isolation of new plant growth-promoting strains *K. pseudosacchari* TL8 and *K. pseudosacchari* TL13. This species belongs to the phylum Proteobacteria, and in particular to the γ-proteobacteria class. This bacterial class, that commonly colonize the rhizosphere of crop plants (Sheridan et al., 2017) or is associated to plant biomass (Montella et al., 2017), is ubiquitous in the soil environment (Ventorino et al., 2019). Indeed, it includes different species that were known to synthesize substances which promote plant growth (i.e., hormones such as IAA, ethylene, and gibberellins), to increase nutrient availability (i.e., N, P, Fe) and their uptake in soil (Kim et al., 2011) and they act as plant disease-suppressive bacteria (Kobayashi et al., 2002; Haas and Défago, 2005). Therefore, the presence of these populations in the soil highlight its high biological fertility potential because they could improve the growth, fitness and health of agricultural plants playing an important role in the bionetwork function of soils (Ventorino et al., 2018). Although many members belonging to the genus *Kosakonia*, as *K. radicincitans*, are known to interact and exert beneficial effects on plant growth (Bergottini et al., 2015; Kämpfer et al., 2016; Berger et al., 2017; Brock et al., 2018), PGP properties in *K. pseudosacchari* species are poorly investigated. Indeed, it was recognized as a novel endophyte species only recently (Kämpfer et al., 2016) and siderophore production was the sole PGP activity previously documented (Arora and Verma, 2017). The main PGP activity by the new PGPR strain *K. pseudosacchari* TL13 was the production of IAA. About 80% of rhizospheric microorganisms are able to produce and release auxins as a secondary metabolite, among these IAA is the most common that can contribute to plant-microbe interaction (Olanrewaju et al., 2017). It is an important growth enhancer because it plays a central role in cell division, elongation, fruit development and senescence, and it has a significant effect on plant root system development (Duca et al., 2014). The concentration of IAA produced by the strain TL13 is similar or higher to that recovered in *K. radicincitans* YD4 strain (about 24 μg mL^–1^) by Bergottini et al. (2015). Interestingly, an increase of 50% of this phytohormone synthesis was observed in the strain grown in the presence of L-tryptophan suggesting a tryptophan-dependent IAA biosynthesis pathway. The synthesis and secretion of IAA could also be linked to the synthesis of ACC synthase in the plant to catalyze the formation of ACC (Glick, 2014). Synthesis of ACC deaminase is also one of the crucial bacterial traits that can facilitate plant growth in the presence of several abiotic or biotic stress (Ali et al., 2014; Glick, 2014). Indeed, *K. pseudosacchari* strains isolated in this work were able to produce ACC deaminase. As for IAA, this is the first work reporting ACC deaminase activity in *K. pseudosacchari* species.

Another interesting PGP activity is the production of siderophores. These are iron-chelating agents with low molecular masses (200–2000 Da), which are produced by microorganisms especially when the bioavailability of Fe is low (Ahmed and Holmström, 2014). Siderophore producing bacteria can improve plant growth by reducing the Fe availability for the phytopathogens and increasing nutrient availability to the plant (Ahmed and Holmström, 2014). As expected, the two *K. pseudosacchari* strains TL8 and TL13 were able to produce iron chelating siderophores, a trait commonly present in *Kosakonia* genus as largely reported by the literature (Arora and Verma, 2017; Chimwamurombe et al., 2016; Lambrese et al., 2018). Siderophore production could be involved also in disease suppression. Indeed, PGPR could act also as biocontrol agents against soil-borne plant pathogens by different ways like competing for nutrients or space, limiting available Fe supply through producing siderophores or by the production of lytic enzymes and antibiosis (Bhattacharyya and Jha, 2012). *K. pseudosacchari* TL8 and TL13 exerted antagonistic activity against *Botrytis* and *Phytophthora* species. To the best of our knowledge, this is the first work reporting suppressive effect against plant pathogens in *K. pseudosacchari* species highlighting that these strains could use also for pest control in agricultural plants.

In addition, *K. pseudosacchari* TL8 and TL13 were also able to solubilize phosphate. Phosphorus is one of the major growth-limiting nutrients required by plants due to its limited availability. There is a great interest in searching phosphate solubilizing bacteria that are able to increase phosphate content and bioavailability in the soil and therefore they are considered promising bio-fertilizers for agriculture enhancement (Kalayu, 2019). Within genus *Kosakonia* this ability was previously reported only in the strain *Kosakonia* sp. A37 (Chakdar et al., 2018).

It is known that some PGPB can fix atmospheric nitrogen into ammonium, and consequently increase the availability of this nutrient in the rhizosphere. The use of these microorganisms in agriculture could decrease the use of chemical N-based fertilizers and therefore their negative impact on the environment as soil quality depletion, pollution and human health (Noar and Bruno-Bárcena, 2018). According to previous works in which several *Kosakonia* species were described as N_2_-fixing bacteria (Chen et al., 2014; Chin et al., 2017; Sun et al., 2018), the new strains *K. pseudosacchari* TL8 and TL13 were able to produce ammonia and potentially able to fix atmospheric nitrogen due to the presence of the *nif*H gene encoding nitrogenase reductase enzyme.

*Kosakonia pseudosacchari* TL8 and *K. pseudosacchari* TL13 showed also interesting abiotic stress tolerance because they were able to grow in a wide range of temperature, pH and salt. These phenotypic properties could help the tolerance of crops cultivated in stress conditions. In particular, salinity is one of the most common abiotic stress in modern agriculture because the irrigation of summer crops with saline water, especially in the coastal regions, lead to an increase of soil salinization in many areas of the world causing major problems for the productivity of agricultural crops and reducing the soil microbial activity (Kumar and Verma, 2018).

As observed *in vitro* assay under fluorescence microscope after treatment with BacLight bacterial viability kit, *K. pseudosacchari* TL8 and TL13 were able to colonize tomato radicles. This result was in according to Kämpfer et al. (2016) which describe *K. pseudosacchari* species as an endophyte of maize plants. Moreover, this ability was also described for other *Kosakonia* species as *K. radicincitans*, able to colonize the root surface of winter wheat (Witzel et al., 2017), or of cucumbers (Sun et al., 2018).

### Production of a Low-Cost and Eco-Sustainable Bacterial Inoculants and Their Effectiveness in Inoculated Plants

Based on PGP traits, the strain *K. pseudosacchari* TL13 was selected for the production of a new low-cost and eco-sustainable bacterial inoculant. In order to develop new bacterial inoculants and to ensure the application of a suitable number of viable and active microbial cells, high biomass production, formulation and shelf life are crucial steps (Bashan et al., 2014). Preliminary investigations in synthetic medium allowed us to assess the growth curve and the best growth condition and parameters to increase bacterial biomass and to obtain a suitable microbial concentration of the strain *K. pseudosacchari* TL13. Besides, microbial cells of TL13 were also subjected to freeze-drying, a common method for preserving bacteria, in order to evaluate their shelf life over time. Although freeze-dried *K. pseudosacchari* TL13 remained viable up to 6 months, this approach could not be suitable at industrial level for its higher production costs than others as foam drying (Morgan et al., 2006). Indeed, production costs of a bio-formulate, which include raw material, equipment and staff, must be competitive in relation to that for the production of chemical fertilizers (Lobo et al., 2019). In general, the use of a low-cost culture medium for the growth and production of microbial biomass is an important issue (Liu et al., 2014; Xu et al., 2015). In this work, to reduce the costs and to develop an eco-sustainable inoculant, the use of several agro-industrial by-products as carbon source was evaluated for the production of *K. pseudosacchari* TL13 by SSF on vermiculite. Indeed, valorization of organic waste biomass and by-products derived from agriculture and food processing factories by a sustainable and harmless disposal have generated interest in microbial biotechnologies (Pagliano et al., 2019). This new approach to by-products management is eco-friendly, easy to be conducted and economically advantageous. Interestingly, *K. pseudosacchari* TL13 was able to use different organic by-products as carbon source although the highest bacterial growth was observed in liquid media containing exhausted yeasts or vinasse. This approach allowed to obtain a suitable bacterial concentration (10^6^ CFU mL^–1^ or g^–1^) in the two final, solid (vermiculite-based) or liquid (raw castor oil/alginate-based emulsion), bio-formulations. The development of two kinds of formulations was important to evaluate their different advantages. Indeed, liquid emulsion formulation allowed to protect the bio−inoculant from desiccation as well as from osmotic and oxidative stress (John et al., 2010); whereas, solid vermiculite-based inoculants were very stable, require no special storage and has good seed-sticking properties (Graham-Weiss et al., 1987). Although, both *K. pseudosacchari* TL13 formulations exerted positive effects on maize plants cultivated in unsterilized soil, the liquid raw castor oil/alginate-based emulsion showed the best results increasing several plant parameters. Liquid formulations are often preferred by users because the product is ready to use. However, the stable and low-cost solid vermiculite-based formulation could be used in agricultural crops for increasing dry matter. These results are in accord with previous works in which inoculum of *Kosakonia* sp. strains were able to exert positive effects in various crops as radish (Berger et al., 2015), yerba mate (Bergottini et al., 2015), tomato (Berger et al., 2017), and maize (Berger et al., 2018).

## Conclusion

The *K. pseudosacchari* strains isolated in this study showed multiple PGP traits as well as antimicrobial activity against several soilborne plant pathogens. In particular, the new selected strain *K. pseudosacchari* TL13 was able to colonize plant roots and improve plant growth. To our knowledge, this is the first work reporting effective multiple PGP abilities and antimicrobial activity in *K. pseudosacchari* species. Moreover, the ability of *K. pseudosacchari* TL13 to efficiently use agro-industrial organic by-products as carbon source for its metabolism makes this strain a promising candidate for the development of new biofertilizers for sustainable agriculture.

## Data Availability Statement

The datasets presented in this study can be found in online repositories. The names of the repository/repositories and accession number(s) can be found at: https://www.ncbi.nlm.nih.gov/genbank/, MN607213 and MN607214.

## Author Contributions

IR performed the experiments, analyzed the data, and drafted the manuscript. VV significantly contributed in results interpretation and drafted the manuscript. PA coordinated the development of bio-formulates and *in vivo* experiments. FC contributed to plant-growth promoting assays and evaluation of microbial growth in liquid media containing agro-industrial by-products. AT provided the soilborne plant pathogens. OP conceived the study and participated in its design and coordination. All authors approved and reviewed the manuscript.

## Conflict of Interest

PA was employed by Agriges S.r.l. The authors declare that this study received funding from Agriges S.r.l. The funder had the following involvement in the study: coordination of the development of bio-formulates and *in vivo* experiments.

## Footnotes

1 http://www.ncbi.nlm.nih.gov/Blast.cgi

2 https://rdp.cme.msu.edu/

3 https://www.ncbi.nlm.nih.gov
